# The association between Western and Prudent dietary patterns and fasting blood glucose levels in type 2 diabetes and normal glucose metabolism in older Australian adults

**DOI:** 10.1016/j.heliyon.2017.e00315

**Published:** 2017-06-07

**Authors:** Erin I. Walsh, Felice N. Jacka, Peter Butterworth, Kaarin J. Anstey, Nicolas Cherbuin

**Affiliations:** a Centre for Research on Ageing, Health and Wellbeing, Australian National University, Canberra, Australia; b Food and Mood Centre, IMPACT Strategic Research Centre, Deakin University, VIC, Australia; c Centre for Adolescent Health, Murdoch Children’s Research Institute, VIC, Australia; d Black Dog Institute, NSW, Australia; e School of Population and Global Health, University of Melbourne, Melbourne, Australia

**Keywords:** Metabolism, Nutrition, Health sciences

## Abstract

High blood glucose and type 2 diabetes are associated with a range of adverse health and cognitive outcomes. One factor that contributes to high blood glucose and type 2 diabetes is dietary intake. This study investigated the relationship between dietary patterns, fasting blood glucose and diabetes status in a sample of 209 participants aged 60–65. Blood plasma glucose was measured from venous blood samples. Individual Prudent and Western dietary patterns were estimated from a self-completed food frequency questionnaire. The relationship between dietary patterns, diabetes, and blood glucose was assessed via general linear model analyses controlling for age, sex, height, and total caloric intake. Results indicated that there was no association between Prudent diet and fasting blood glucose levels, or type 2 diabetes. In contrast, an individual in the upper tertile for Western dietary score had a significantly higher risk of having diabetes than an individual in the lower tertile for Western dietary score. However, there was no significant association between Western diet and fasting blood glucose. Western diet may be associated with type 2 diabetes through mechanisms beyond impacting blood plasma glucose directly. The fact that the association between Western diet and type 2 diabetes remained even when total caloric intake was controlled for highlights the need for policy and population health interventions targeting the reduction of unhealthy food consumption.

## 1 Introduction

Type 2 diabetes is a substantial and growing contributor to the global disease burden [1]. It is associated with a wide range of negative health outcomes, including cognitive decline, dementia, and cardiovascular disease [2, 3]. Even within the range of normal glucose metabolism (fasting blood plasma glucose concentration of <5.6 mmol/L, [4]), high blood glucose levels are a major risk factor for the onset of type 2 diabetes [5], and are also associated with adverse health outcomes, including dementia, ischaemic heart disease and stroke [6, 7, 8]. Because of the relationship between high blood glucose and health in both individuals with normal blood glucose and those with diabetes, it is important to better understand the factors that influence blood plasma glucose levels, and the onset of type 2 diabetes. This is particularly so in the context of ageing, as glucose metabolism becomes less efficient, and consequently blood glucose levels tend to increase, as people age [9, 10].

Diet is a key factor which contributes to blood glucose levels. Dietary management can lower blood glucose levels in those with [11] and without type 2 diabetes [12]. Long-term diet is associated with increased type 2 diabetes incidence [13, 14]. However, the extent to which dietary characteristics drive these effects is not understood. Energy density and nutritional quality of food have somewhat independent impacts on health [15]. Higher caloric intake is associated with increased blood glucose and type 2 diabetes incidence [16], but the impact of the types of food consumed on blood glucose and type 2 diabetes incidence is more unclear. Past research has operationalised the human diet in a number of ways, such as by examining specific food groups [13], or deconstructing of the whole diet into individual nutrient groups [12]. While such reductionist approaches have proven somewhat useful they have several limitations. A focus on food type or nutrient group in isolation can lead to erroneous conclusions which do not necessarily take into consideration how food is prepared or how food types are combined. In real-world settings, people eat meals consisting of a mixture of nutrient groups, which often have interactive effects on digestion and health [17]. An alternative approach which reflects the multi-nutrient, mixed nature of meals is dietary pattern analysis.

Rather than focussing on a particular food group, dietary pattern analysis is conceptually attractive as it takes into account the whole diet, and so accounts for the synergistic effects of nutrients on health. As described in Hu [17], this approach typically identifies two main dietary patterns: a ‘Western’ dietary pattern (characterised by processed foods, and foods high in sugars and fats), and a ‘Prudent’ dietary pattern (characterised by fruit, vegetables, lean meat, fish and unprocessed grains). Because an individual may choose any combination of foods within the bounds of total caloric intake (the total amount of energy from food an individual consumes), these dietary patterns are not mutually exclusive and generally are only marginally (inversely) correlated. Moreover their associations with health outcomes are commonly independent of one another [18]. One shortcoming of the dietary pattern analysis approach is that the patterns extracted are somewhat dependent on the analytical process used in their calculation [19, 20]. Nonetheless, dietary pattern analysis is sufficiently robust to support a growing literature examining the relationship between diet and health.

A number of cross-sectional and longitudinal studies have used dietary pattern analysis and found that the Prudent dietary pattern is associated with lower fasting blood glucose and a lower incidence of type 2 diabetes, while the Western dietary pattern has been linked to higher fasting blood glucose and higher incidence of type 2 diabetes [21, 22]. Studies that have failed to find an association between Prudent or Western dietary patterns and blood glucose (i.e.[23]) explicitly excluded individuals on the basis of diabetes diagnosis, including either only those with or without diabetes. Doing so may have obscured a continuous relationship between dietary pattern and blood glucose by truncating the range of blood glucose according to diabetes diagnosis.

The aim of the present study was to investigate the relationship between Prudent and Western dietary patterns and fasting blood glucose, considering participants across the full range of blood glucose levels (those without diabetes as well as those with impaired fasting glucose or type 2 diabetes), and to examine whether the association is changed when stratifying analyses on the basis of impaired fasting glucose and type 2 diabetes diagnosis. Further investigation of the relationship between diet and impaired fasting glucose or type 2 diabetes incidence will help clarify if there are categorical differences in dietary patterns between individuals with blood glucose in the normal range, with impaired fasting glucose or with type 2 diabetes which may underlie differences in blood plasma glucose.

## 2 Materials and methods

### 2.1 Study population

The sample for this study included participants from the PATH Through Life project, a longitudinal study of ageing described elsewhere [24]. Briefly, participants were residents of the cities of Canberra and Queanbeyan, recruited randomly through the electoral roll. Enrolment to vote is compulsory for Australian citizens. This investigation focuses on the Wave 1 oldest cohort (N = 2551), aged 60–64 at baseline when the dietary data were collected. Of these participants, 478 were randomly selected and invited to take part in the MRI sub-study, which involved a blood assessment. Blood glucose was available for 271. From the 262 of those with available dietary data, a further 54 were excluded due to self-reported history of stroke, epilepsy, or low cognition (as measured an MMSE score of <25; [25]), leaving data on 208 participants available for analyses.

### 2.2 Standard protocol approvals, registrations, and patient consents

The study was approved by the Australian National University Ethics Committee and all participants provided written informed consent.

### 2.3 Dietary measures

The Commonwealth Scientific and Industrial Research Organisation Food Frequency Questionnaire was used to measure dietary intake. Validated for use in an Australian population, this measure includes food types, serving sizes, cooking methods and general eating habits [26]. Scores for Western and Prudent dietary patterns were calculated from the Food Frequency Questionnaire. As described elsewhere [18], daily grams of a total of 188 food items consumed were extracted from the Food Frequency Questionnaire, and summarised into two orthogonal dietary pattern factors (Prudent/Western) using Principle Components Analyses. The dietary pattern variables used in the current study were the z scores of the continuous factor loadings for each participant, and binned into tertiles to allow comparison of low, centre, and high scoring individuals. A Prudent dietary pattern is characterised by fresh fruit, vegetables, grilled fish and salad, while a Western dietary pattern is characterised by sausages, roast meat, chips and crisps, and soft drinks.

### 2.4 Blood glucose

Venous blood was collected after a skipped breakfast. Plasma glucose was measured on an LX20 analyser by an oxygen rate method (Beckman-Coulter, Fullerton, CA). Individuals had either normal blood glucose (blood glucose <5.6 mmol/L), impaired fasting glucose (blood glucose 5.6–<7 mmol/L), or type 2 diabetes (blood glucose> = 7 mmol/L or self-reported as having diabetes).

### 2.5 Statistical analysis

Multinomial logistic regression was then used to examine the relationship between dietary score tertile and blood glucose as a continuous variable, as well as blood glucose as a continuous variable. Where appropriate, the lowest tertile group and individuals with normal blood glucose were used as the base group for comparisons. Linear models were then used to investigate the association between dietary tertiles and continuous blood glucose levels. Adjusted models controlled for age, gender, height, and total energy intake. Height was controlled for to take into account body size while not over-correcting by using related measures such as body mass index (however, sensitivity analyses controlling for BMI were also carried out). Total energy intake was centred on the sample mean. One individual with very high fasting blood glucose (16.1 mmol/L) was removed from analysis as their data comprised an influential outlier (Cook’s distance >1), and destabilised coefficients in sensitivity analyses. Alpha was set at 0.05.

## 3 Results

### 3.1 Descriptive characteristics

**Fig. 1:**
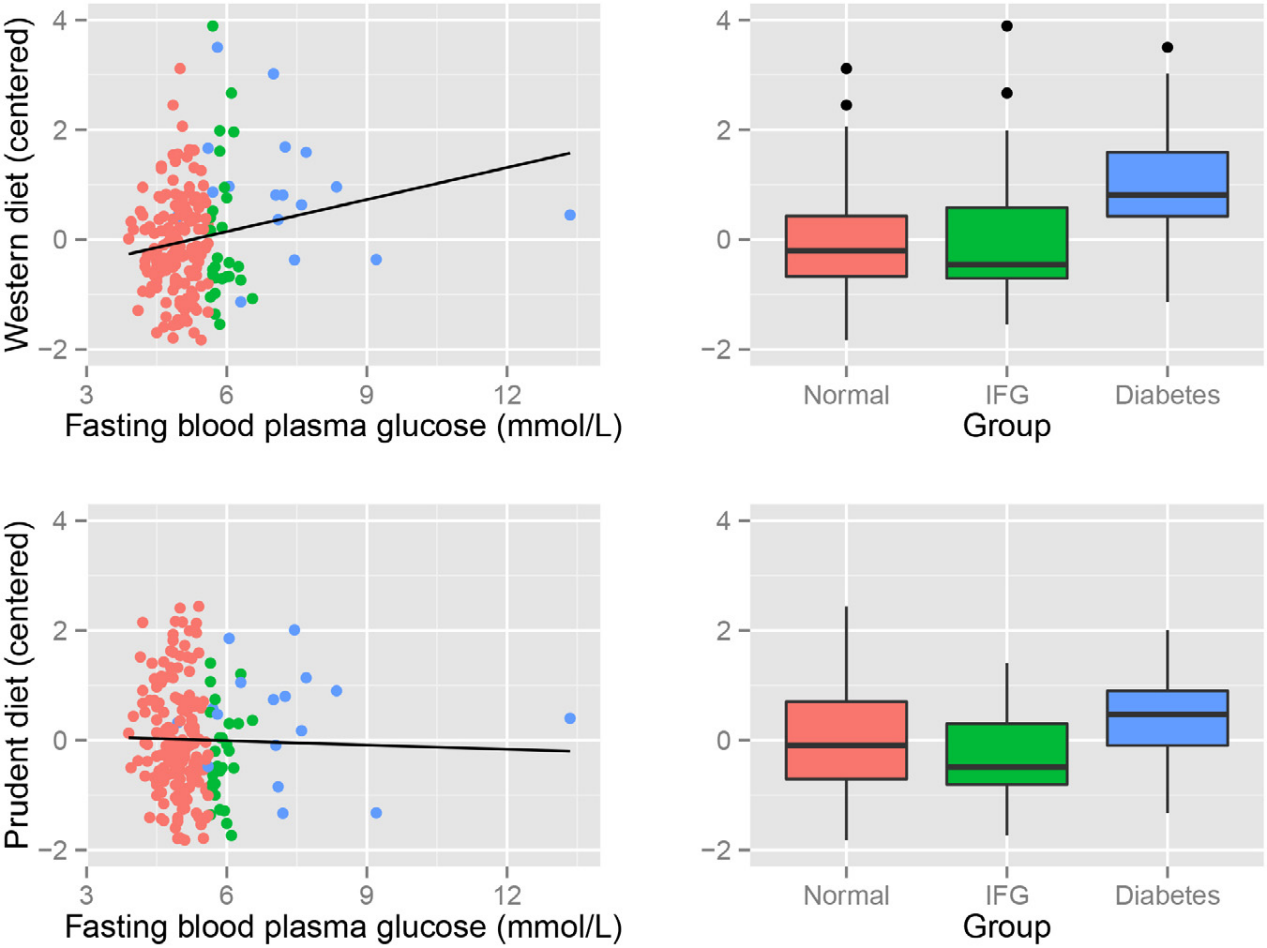
Association between dietary patterns and fasting blood plasma glucose. Note. Fitted lines show linear trends which do not control for covariates used in modelling, hence energy intake having a negative slope in models but positive slope pictured here. The outlier excluded from analysis is not pictured.

Participant demographic characteristics can be found in Table 1. Diabetes and IFG prevalence in the current sample (8.2% and 13% respectively) were slightly higher than contemporary estimates for 2000 in Australia (e.g. AusDiab for the age bracket of 55–64 estimates 6.5% and 8.7% [27]). Fasting blood plasma glucose ranged from 3.9 to 13.35 mmol/L (mean 5.25 mmol/L, SD = 0.93). The continuous association between dietary patterns and blood glucose was diffuse (Fig. 1). Dietary score tertiles included ≈69 per group, with Western dietary pattern z scores binned as low [-1.83,-0.488], center (-0.488,0.353], and high (0.353,3.11], and Prudent scores binned as low [-2.11,-0.543], center (-0.543,0.377], and high (0.377,2.16].

**Table 1:** Participant demographic characteristics.

	All (n = 208)	Normal range (n = 163)	Impaired Fasting Glucose (n = 28)	Type 2 Diabetes (n = 17)
Age	63.27 (1.39) [60.77, 65.98]	63.28 (1.44) [60.77, 65.98]	63.14 (1.31) [61.11, 65.81]	63.49 (1.03) [61.96, 65.46]
Gender	109 (52%) male	84 (51%) male	14 (50%) male	11 (65% male)
Height (cm)	170.33 (9.46) [148.00, 193.00]	170.33 (9.38) [149.86, 193.00]	168.99 (8.9) [148.00, 182.88]	172.57 (11.21) [149.86, 185.42]
Body Mass Index	26.17 (4.23) [17.20, 42.06]	25.15 (3.53) [17.20, 40.63]	29.03 (4.04) [22.01, 37.18]	31.41 (4.91) [23.32, 42.06]
Total energy intake (kj)	8235.85 (2171.5) [3630.00, 15658.68]	8091.07 (2081.32) [3630.00, 15658.68]	8325.72 (2469.17) [4563.25, 13097.98]	9476.03 (2234.92) [5744.68, 13425.98]
Blood Glucose (mmol/L)	5.25 (0.94) [3.90, 13.35]	4.93 (0.39) [3.9, 5.6]	5.89 (0.23) [5.65, 6.55]	7.27 (1.90) [4.95, 13.35]

Figure in brackets is standard deviation, in square brackets is range.

### 3.2 Dietary tertiles, diabetes, and IFG

A prudent score tertile was not significantly associated with either diabetes or IFG (Table 2). Individuals in the highest western score tertile were fourteen times more likely to have diabetes than those in the lowest score tertile (in the low tertile, diabetes prevalence was 1%, medium was 3% and high was 19%), and those in the medium tertile were significantly less likely to have IFG (in the low tertile, IFG prevalence was 20%, in the middle tertile 7%, and in the high tertile 11%). Coefficient directionality and significance was unaffected by controlling for age, gender, height, and energy intake. These results suggested that subsequent analyses should subdivide analyses by diabetes group, specifically NFG and non-NFG (including diabetes and IFG to preserve sample size). Bonferroni correction to account for multiple comparisons (repeating whole sample analysis with sub-sample, so 0.05/3) set alpha to 0.016.

**Table 2:** Multinomial model associations between dietary tertiles and diabetes category.

	Diabetes						IFG				
			RR						RR		
	b	p	Est	L	U		b	p	Est	L	U
Unadjusted											
(Intercept)	-4.38	< 0.01*	0.01	0	0.12		-1.16	< 0.01*	0.31	0.14	0.68
Prudent (center)	0.17	0.83	1.19	0.24	5.85		-0.02	0.97	0.98	0.39	2.49
Prudent (high)	0.88	0.23	2.4	0.58	9.91		-0.78	0.18	0.46	0.15	1.43
Western (center)	0.58	0.64	1.78	0.16	20.26		-1.12	0.04*	0.33	0.11	0.97
Western (high)	2.65	0.01*	14.16	1.77	113.02		-0.34	0.49	0.71	0.27	1.86
Adjusted											
(Intercept)	-27.25	0	0	0	0		3.47	0	32.06	31.06	33.08
Prudent (center)	0.08	0.93	1.08	0.2	5.88		-0.27	0.6	0.76	0.28	2.09
Prudent (high)	0.93	0.25	2.55	0.52	12.38		-1.16	0.07	0.31	0.09	1.09
Western (center)	0.23	0.85	1.26	0.1	15.25		-1.38	0.02*	0.25	0.08	0.82
Western (high)	2.49	0.02*	12.08	1.37	106.65		-0.87	0.16	0.42	0.12	1.43
Age	0.37	0	1.45	1.19	1.78		0	0.95	1	0.85	1.18
Gender (female)	-0.24	0.78	0.79	0.15	4.13		0.03	0.96	1.03	0.31	3.47
Height	0	0.91	1	0.93	1.07		-0.03	0.37	0.97	0.92	1.03
Energy Intake	0.23	0.49	1.25	0.66	2.39		0.49	0.08	1.63	0.95	2.81

RR = Risk Ratio, L and U refer to corresponding 95% upper and lower confidence intervals.* indicates significance at α < 0.05.

### 3.3 Dietary tertiles and blood glucose as a continuous variable

When all participants were included in the model, prudent diet was not significantly associated with fasting glucose (Table 3). In the unadjusted model, the highest tertile for Western diet score was significantly higher blood glucose, such that individuals in that group had 0.43 mmol/L higher blood glucose than those in the low tertile, though significance was lost in the adjusted model. Neither Prudent nor Western pattern was significantly associated with blood glucose levels when the sample was divided into NFG only, and non-NFG (IFG and diabetes) only.

**Table 3:** Linear model associations between dietary tertile and fasting blood glucose.

	Unadjusted					Adjusted			
	b	p	L	U		b	p	L	U
All participants									
(Intercept)	5.17	< 0.01*	4.89	5.45		3.10	0.38	-3.92	10.12
Prudent (center)	-0.10	0.52	-0.41	0.21		-0.05	0.75	-0.39	0.28
Prudent (high)	-0.02	0.88	-0.34	0.29		0.03	0.87	-0.32	0.38
Western (center)	-0.08	0.64	-0.39	0.24		-0.12	0.47	-0.46	0.21
Western (high)	0.43	0.01*	0.11	0.74		0.36	0.06	-0.02	0.73
Age						0.02	0.70	-0.08	0.11
Gender (female)						-0.12	0.55	-0.53	0.28
Height						0.01	0.59	-0.01	0.03
Energy Intake						< 0.01	0.97	-0.16	0.17
									
NFG only									
(Intercept)	4.99	< 0.01*	4.85	5.12		6.31	< 0.01*	3.03	9.59
Prudent (center)	-0.09	0.24	-0.23	0.06		-0.04	0.59	-0.20	0.11
Prudent (high)	-0.18	0.02	-0.33	-0.03		-0.13	0.10	-0.30	0.03
Western (center)	-0.02	0.79	-0.16	0.12		-0.03	0.65	-0.18	0.12
Western (high)	0.12	0.12	-0.03	0.28		0.06	0.53	-0.12	0.23
Age						-0.02	0.30	-0.07	0.02
Gender (female)						-0.15	0.13	-0.33	0.04
Height						< 0.01	0.80	-0.01	0.01
Energy Intake						0.01	0.71	-0.06	0.09
									
Diabetes and IFG only									
(Intercept)	5.88	< 0.01*	5.02	6.73		-7.06	0.55	-30.75	16.63
Prudent (center)	-0.14	0.78	-1.12	0.84		0.19	0.71	-0.86	1.24
Prudent (high)	0.65	0.21	-0.36	1.66		1.27	0.04	0.06	2.48
Western (center)	0.43	0.49	-0.79	1.65		0.68	0.31	-0.67	2.03
Western (high)	0.69	0.14	-0.21	1.58		0.76	0.21	-0.45	1.98
Age						0.14	0.44	-0.23	0.50
Gender (female)						-0.57	0.39	-1.90	0.75
Height						0.02	0.42	-0.04	0.09
Energy Intake						-0.45	0.08	-0.96	0.05

L and U refer to 95% upper and lower confidence intervals for the slope.* indicates significance at α < 0.016.

### 3.4 Sensitivity analyses

To maximise power, multinomial models were repeated, comparing individuals with blood glucose in the normal range against those with IFG or diabetes (combining the two groups). This indicated no significant association between IFG and diabetes and Prudent dietary tertiles (mid estimate = −0.18, *p* = 0.68, RR = 0.83, 95%CI[-1.08, 0.71]; high estimate = −0.32, *p* = 0.50, RR = 0.72, 95%CI[-1.27,0.62]), and a pattern similar to the previously reported association between Western dietary tertiles and IFG (mid estimate = −1.10, *p* = 0.03, RR = 0.33, 95%CI[0.12, 0.93]; high estimate = 0.17, *p* = 0.72, RR = 1.19, 95%CI[0.46, 3.05]). When adjusting for BMI rather than height, coefficient directionality and significance for prudent dietary tertiles were unaffected. However, the high Western tertile gained significance for IFG (estimate = -1.46, p = 0.04, RR = 0.231, 95%CI[-2.89, −0.03]). In linear analyses, no dietary tertile was significantly associated with blood glucose when analyses were limited to individuals with diabetes only, though results are likely unstable due to small cell sizes. Analyses considering dietary pattern scores as continuous variables (Table 4, Table 5 and Table 6) demonstrated a similar pattern of conclusions.

**Table 4:** Linear models of Prudent dietary pattern (continuous) and fasting blood glucose.

	Fasting blood glucose			
	All	Normal range	IFG	T2D
	(1)	(2)	(3)	(4)
Age	-0.027 (0.037)	-0.033 (0.021)	0.010 (0.034)	-0.069 (0.353)
Gender	-0.112 (0.159)	-0.29 (0.093)	-0.030 (0.128)	0.418 (1.417)
Total Energy Intake	0.155* (0.059)	0.024 (0.036)	0.048 (0.043)	0.347 (0.534)
Height	-0.003 (0.008)	0.002 (0.005)	-0.020* (0.007)	< 0.01 (0.068)
Prudent diet	-0.070 (0.059)	-0.039 (0.034)	-0.073 (0.065)	-0.407 (0.518)
Constant	7.56* (2.756)	6.687* (1.585)	8.670* (2.278)	11.116 (28.285)
Observations	207	163	28	16
R^2^	0.054	0.082	0.333	0.125
Adjusted R^2^	0.031	0.053	0.181	-0.313
Residual Std. Error	0.737 (df = 201)	0.327 (df = 157)	0.205 (df = 22)	1.267 (df = 10)
F Statistic	2.304* (df = 5; 201)	2.796* (df = 5; 157)	2.192* (df = 5;22)	0.286 (df = 5; 10)

Values reported are slopes (*b*). Values in brackets are standard errors. Significance at α < 0.05 indicated by *; df: degrees of freedom.

**Table 5:** Linear models of Western dietary pattern (continuous) and fasting blood glucose.

	Fasting blood glucose			
	All	Normal range	IFG	T2D
	(1)	(2)	(3)	(4)
Age	-0.027 (0.037)	-0.034 (0.021)	0.023 (0.032)	-0.209 (0.325)
Gender	-0.146 (0.151)	-0.162 (0.090)	-0.070 (0.125)	-1.007 (0.931)
Total Energy Intake	0.057 (0.064)	0.007 (0.038)	0.012 (0.051)	0.706 (0.527)
Height	-0.003 (0.008)	0.002 (0.005)	-0.019* (0.007)	-0.047 (0.058)
Western diet	0.094 (0.064)	-0.004 (0.041)	0.030 (0.045)	-0.514 (0.305)
Constant	7.454* (2.750)	6.853* (1.588)	7.638* (2.060)	28.672 (25.638)
Observations	207	163	28	16
R^2^	0.058	0.074	0.309	0.277
Adjusted R^2^	0.034	0.045	0.152	-0.085
Residual Std. Error	0.736 (df = 201)	0.381 (df = 157)	0.209 (df = 22)	1.152 (df = 10)
F Statistic	2.463* (df = 5; 201)	2.511* (df = 5;157)	1.964 (df = 5; 22)	0.765 (df = 5; 10)

Values reported are slopes (*b*). Values in brackets are standard errors. Significance at α < 0.05 indicated by *; df: degrees of freedom.

**Table 6:** Models with continuous dietary scores, controlling for BMI, rather than height.

	Fasting blood glucose			
	All (n = 208)	Normal Range (n = 163)	IFG (n = 28)	T2D (n = 17)
	(1)	(2)	(3)	(4)
Age	0.037	-0.030	-0.009	-0.374
	(0.046)	(0.022)	(0.037)	(0.750)
				
Gender	-0.197	-0.119	0.144	-2.432
	(0.145)	(0.067)	(0.116)	(1.760)
				
BMI	0.088^**^	0.018	-0.007	0.022
	(0.016)	(0.009)	(0.017)	(0.119)
				
Western diet	0.044	-0.014	0.010	-0.085
	(0.085)	(0.043)	(0.050)	(0.841)
				
Prudent diet	-0.017	-0.049	0.003	0.644
	(0.073)	(0.034)	(0.090)	(0.821)
				
Total energy intake	0.006	0.017	0.019	-0.820
	(0.088)	(0.043)	(0.060)	(0.920)
				
Constant	0.698	6.431^**^	6.507	31.672
	(3.046)	(1.429)	(2.579)	(47.732)

Observations	193	152	25	16
R^2^	0.177	0.100	0.096	0.216
Adjusted R^2^	0.151	0.063	-0.206	-0.307
Residual Std. Error	0.880 (df = 186)	0.374 (df = 145)	0.208 (df = 18)	2.194 (df = 9)
F Statistic	6.680^***^ (df = 6; 186)	2.693^**^ (df = 6; 145)	0.317 (df = 6; 18)	0.413 (df = 6; 9)

Values reported are slopes (b). Values in brackets are standard errors. Significance at α < 0.05 indicated by *; df: degrees of freedom.

## 4 Discussion

This study investigated the relationship of Prudent and Western dietary patterns with fasting blood glucose and diabetes status. Regardless of whether analysis included the full range of blood glucose levels, or were stratified on the basis of normal blood glucose, impaired fasting glucose, and type 2 diabetes, neither Prudent nor Western dietary patterns were significantly associated with fasting blood glucose. This is consistent with findings by Esmailzadeh and colleagues [23], and suggests that the lack of significant association between dietary pattern and blood glucose in their study may not reflect the truncation of the full blood glucose range by excluding or controlling for diabetes status as hypothesised.

Congruent with Cordain et al. [21], those with type 2 diabetes were significantly more likely to be in the highest Western diet tertile than those with blood glucose in the normal range which supports the view that, at least in part, past diet contributes to current glucose levels/metabolic status. Importantly, the association between Western dietary pattern and type 2 diabetes was detected after controlling for caloric intake. This suggests the quality of the Western diet beyond its generally increased caloric content is likely to explain diabetes diagnosis, and that it is likely this is associated with adiposity. A Western diet has been associated with insulin resistance in correlational human studies and experimental animal models even when, as in the current study, caloric intake is controlled for [23]. Identification of the possible mechanisms underlying this association are beyond the scope of this study, but previous research suggests two parallel pathways.

Firstly, diet has a direct influence on insulin resistance and type 2 diabetes [28]. As well as causing repeated transient spikes in blood glucose, the western diet is a pro-inflammatory diet and has been shown to be associated with increases in a variety of inflammatory biomarkers and cytokines (CRP, IL1, IL6) and increased oxidative stress which together lead to cell and DNA damage, decrease in insulin receptor numbers and lower insulin production [28]. Secondly, the Western diet is associated with other type 2 diabetes comorbidities, such as a higher BMI and hyperlipidaemia [5]. These in turn may increase type 2 diabetes risk through mechanisms whose effects would not necessarily be detected by measuring fasting blood glucose, such as causing dysfunction in pancreatic cells that secrete insulin [29]. A future focus of research should therefore be to explore these possibilities by recording concurrent known type 2 diabetes risk factors alongside diet, measuring blood glucose in a way that would detect transient spikes in blood glucose (such as sampling following meals), and examining blood for inflammatory markers.

These explanations assume that diet has a unidirectional impact on type 2 diabetes, but another consideration is the impact of type 2 diabetes on diet. Following diabetes diagnosis, an individual is typically advised to make a number of lifestyle adjustments, including dietary and physical exercise modification, alongside medication to manage blood glucose [11]. Similar lifestyle adjustment from long-term unhealthy diet may also explain the significantly higher number of individuals with IFG in the lowest Western diet tertile, as opposed to the central one. The assumption that type 2 diabetes onset and subsequent elevated blood glucose levels are caused by the same factors requires scrutiny. Specifically, the relationship between the Western diet and fasting blood plasma glucose in type 2 diabetes could be masked by post-diagnosis lifestyle adjustments, and medication, in individuals with diabetes. Future research could investigate this possibility by longitudinally examining diet and other lifestyle factors to see if they predict type 2 diabetes onset, and then investigating whether type 2 diabetes onset is associated with lifestyle change. Comparison of individuals with diabetes based on whether or not they are medicated would be particularly illuminating.

Contrary to previous studies [22], the Prudent dietary pattern was not associated with IFG or type 2 diabetes. This supports the partly orthogonal nature of the relationship between the two dietary patterns. If they were orthogonal, the higher Western diet in participants with type 2 diabetes would necessarily also be associated with a lower Prudent diet in that sample. It further suggests that a Prudent dietary pattern is not sufficiently protective to thwart the mechanisms by which the Western diet is associated with type 2 diabetes. Previous research suggests that each dietary pattern is an independent predictor of health outcomes and that the patterns do not interact to jointly influence such outcomes (e.g. [18]).

This study has some limitations, and some significant strengths. Causality cannot be established due to the cross-sectional design. Participants in the MRI subsample for whom the glucose data were available were a random subsample from a larger randomly sampled group. This is beneficial for representativeness of the wider community, so resulted in a small number of individuals with IFG and diabetes. Further, the original sample may have been somewhat biased due to selection effects, and the requirement of participant literacy. It is possible that an association between diet and blood glucose exists, but was not found in the current study due to insufficient statistical power or insensitive measures. The current study did not collect a second blood glucose measurement, in particular to confirm high blood glucose, and so undetected measurement errors may have distorted results. Fasting blood glucose provides a relatively short-term insight into blood glucose levels compared with HbA1c, which assesses average plasma blood glucose in the 120 days preceding measurement, (weighted somewhat toward the more recent plasma blood glucose levels) [30]. The use of repeated fasting glucose measurement, HbA1c, or a combination of techniques, would add precision to future research investigating the association between diet and blood glucose levels. The operationalisation of dietary patterns in the current study may also have been imprecise, due to reliance on self-reported food intake. Compared with direct observation of food purchase, self-report is vulnerable to social desirability effects, typically causing an underreporting of food intake [31]. This may have resulted in an erroneously constricted range of self-reported food intake by truncating particularly high dietary pattern scores, and potentially masking a relationship between very high dietary pattern scores and blood glucose. Additionally, although using principal component analysis for dietary pattern extraction is standard practise, it can produce variable results depending on analytical choices made during their calculation [20]. This could be further investigated, as in Ashby-Mitchel, Peeters and Anstey [19], to see if conclusions remain consistent when different analytical choices are made when creating dietary pattern scores.

## 5 Conclusions

In conclusion, this study found no association between a Prudent dietary pattern and blood glucose levels, or type 2 diabetes. The pattern of results suggests that a Western dietary pattern is associated with type 2 diabetes and that this effect may either be due to sustained dietary intake in the past or through mechanisms that do not directly affect blood plasma glucose. Because of the well-documented health impacts of type 2 diabetes diagnosis, the nature of these mechanisms requires further investigation. The fact that the Prudent diet did not have a protective effect for blood glucose levels or type 2 diabetes, and that the association between Western dietary pattern and type 2 diabetes remained significant even after total caloric intake was controlled for, highlights the need for policy and population health interventions to initiate behaviour change specifically targeted at reducing unhealthy food consumption in addition to those aimed at reducing excessive caloric intake.

## Declarations

### Author contribution statement

Erin I. Walsh: Performed the experiments; Analyzed and interpreted the data; Wrote the paper.

Felice N. Jacka, Peter Butterworth: Conceived and designed the experiments; Performed the experiments; Analyzed and interpreted the data.

Kaarin J. Anstey: Conceived and designed the experiments; Contributed reagents, materials, analysis tools or data.

Nicolas Cherbuin: Conceived and designed the experiments; Performed the experiments; Contributed reagents, materials, analysis tools or data.

### Funding statement

This work was supported by the National Health and Medical Research Council (grant numbers 229936, 179839, 973302 and 157125). Nicolas Cherbuin and Kaarin Anstey’s Research Fellowship are funded by the Australian Research Council (grant number 120100227) and the National Health and Medical Research Council (grant number 1002560). Felice Jacka has received Grant/Research support from the Brain and Behaviour Research Institute, the National Health and Medical Research Council (NHMRC), Australian Rotary Health, the Geelong Medical Research Foundation, the Ian Potter Foundation, Eli Lilly, the Meat and Livestock Board, Woolworths Limited, Fernwood Gyms and The University of Melbourne and has received speakers honoraria from Sanofi-Synthelabo, Janssen Cilag, Servier, Pfizer, Health Ed, Network Nutrition, Angelini Farmaceutica, Eli Lilly and Metagenics. She is supported by an NHMRC Career Development Fellowship (2) (#1108125).

### Competing interest statement

The authors declare no conflict of interest.

### Additional information

No additional information is available for this paper.
